# RAR‐related orphan receptor A: One gene with multiple functions related to migraine

**DOI:** 10.1111/cns.13453

**Published:** 2020-09-05

**Authors:** Sedigheh Farahani, Leila Solgi, Sahar Bayat, Atieh Abedin Do, Shohreh Zare‐Karizi, Behnam Safarpour Lima, Reza Mirfakhraie

**Affiliations:** ^1^ Department of Genetics School of Biological Sciences Varamin‐Pishva Branch Islamic Azad University Varamin Iran; ^2^ Department of Medical Genetics Faculty of Medicine Tabriz University of Medical Sciences Tabriz Iran; ^3^ Department of Medical Genetics School of Medicine Shahid Beheshti University of Medical Sciences Tehran Iran; ^4^ Department of Regenerative Medicine Faculty of Medicine GREB Dental Faculty Laval University Quebec Canada; ^5^ Department of Neurology School of Medicine Shahid Beheshti University of Medical Sciences Tehran Iran; ^6^ Genomic Research Centre Shahid Beheshti University of Medical Sciences Tehran Iran

**Keywords:** genetic association study, migraine, *RORA*, rs11639084, rs4774388

## Abstract

**Aims:**

RAR‐related orphan receptor (*RORA*) involves in regulation of several biological processes including inflammation and circadian rhythm that probably are involved in migraine pathophysiology. In the current study, the association between *RORA* rs11639084 and rs4774388 variants and susceptibility to migraine were investigated in a sample of Iranian migraine patients for the first time.

**Methods:**

In a case‐control study including 400 participants, 200 migraineurs and 200 healthy controls, genotyping of *RORA* rs4774388 and rs11639084 polymorphisms was performed using tetra‐primer amplification refractory mutation system–polymerase chain reaction (TP‐ARMS‐PCR).

**Results:**

The distribution of rs4774388 C/T and T/T genotypes differed significantly between the studied groups. Moreover, an association was observed between rs4774388 and migraine under the recessive mode of inheritance (*P* = 0.002; OR = 1.89.; CI = 1.25‐2.87). The distribution of rs11639084 alleles and genotypes was not significantly different between migraineurs and healthy controls.

**Conclusion:**

Current results suggest *RORA*, as a molecular link, may explain inflammation and circadian rhythm dysfunction in migraine. Further studies in different ethnicities are required to confirm the function of *RORA* in migraine development.

## Graphical abstract

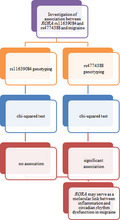

## 1 INTRODUCTION

Migraine is the most common paroxysmal neurological disorder with a polygenic multifactorial inheritance.^1^ The disease affects approximately up to 15% of the adult population and also is more common among women.^2^ It can cause severe episodic headache, autonomic dysfunction and deregulation of neurological reactions.^3^ Epidemiological studies have demonstrated that patients with a positive family history carry an increased hereditary risk for the disease.^2^

The pathophysiology of migraine is still unknown; nevertheless, a significant comorbidity has been reported between migraine and allergic diseases such as asthma and seasonal allergies that highlights evidence of immune involvement in migraine.^4^, ^5^, ^6^, ^7^ Based on some hypotheses, the development and progression of this disorder may be affected by inflammation, vascular dysfunction, circadian rhythm, and several signaling pathways.^8^, ^9^, ^10^ Evidence shows a positive association between the elevated inflammatory agents and migraine disorder.^11^ Among these agents, cytokines and CD4^+^ T cells are considered to play an essential role in neurovascular inflammation.^12^ Moreover, several findings suggested a sharp increase in plasma T‐helper 17 (Th17) cells, a member of three subsets of CD4^+^ T cells, and effector cytokines levels including IL‐4, IL‐5, IL‐6, IL‐10, and IL‐17A during neurovascular inflammation among migraine patients.^13^, ^14^, ^15^ Th17 cells secrete IL‐17 that induces chronic inflammatory disorders.^16^, ^17^, ^18^, ^19^ Remarkably, the above‐mentioned cytokines, especially IL‐10, play essential roles in Th17 differentiation.^20^

Although the genetic basis of migraine is not clear yet, several susceptibility regions, including the 15q11‐q13, have been suggested to be associated with migraine.^21^ Retinoic acid receptor‐related orphan receptor alpha (*RORA*) is one of the three ROR nuclear receptors (α, β, and γ) located at 15q22.2 and is expressed in several tissues, including brain, lungs, liver, skeletal muscle, kidney, and thymus.^10^, ^22^ *RORA* plays a key role in the regulation of Th17‐cell differentiation, circadian rhythms, and inflammation.^23^, ^24^ Several studies have demonstrated that *RORA* could regulate numerous cytokine expressions and Th17 differentiation directly or indirectly.^24^, ^25^, ^26^ Induction of *RORA* expression by TGFβ and IL‐6 results in the differentiation of Th17 cells as well as expression of IL‐17.^24^ Moreover, RORA itself can trans‐activate the expression of IL‐6 which also plays a role in the Th17‐cell differentiation.^25^, ^26^

Based on the above‐mentioned considerations, we hypothesized that the *RORA* gene might be a probable candidate gene involved in migraine pathophysiology. Therefore, in this study, for the first time, we aimed to investigate the association of *RORA* rs11639084 and rs4774388 variants with migraine susceptibility in Iranian patients.

## 2 MATERIALS AND METHODS

### 2.1 Subjects

In the present case‐control study, 400 subjects, including 200 migraine patients and 200 healthy controls, were enrolled and referred from Imam Hossein hospital, Tehran, Iran, between December 2014 and December 2015. Blood sample collection was performed from all subjects in EDTA tubes. Migraine diagnosis was confirmed according to the International Classification of Headache Disorders criteria^27^ based on the brain imaging studies and neurological examination. Healthy participants did not suffer from chronic headache and neurological diseases. The age and sex were matched in the studied groups. The mean age was 34.98 ± 6.81 years and 34.23 ± 7.12 years in the patients and controls, respectively. Demographic and clinical characteristics of the migraine patients are shown in Table 1. The Ethics Committee of the Shahid Beheshti University of Medical Sciences (SBMU) approved the study protocol (Code No: IR.SBMU. MSP.REC.1397.702), and all participants or their parents signed the informed written consent.

**Table 1:** Demographic and clinical data of the migraine patients

Migraine patients	
Parameter	N (%)
Gender	
Male	47 (23.5)
Female	153 (76.5)
Type	
MA	58 (29)
MO	142 (71)
Location	
BL	108 (54)
UL	92 (46)
Nausea/Vomiting	
Yes	157 (78.5)
No	43 (21.5)

Abbreviations: BL, Bilateral; MA, Migraine with aura; MO, Migraine without aura; UL, Unilateral.

### 2.2 Genotyping of *RORA* rs11639084 and rs4774388 variants

Genomic DNA was isolated from blood samples using the GeneAll Exgene™ Blood SV Kit (GeneAll Biotechnology, Seoul, Korea) based on the manufacturer's protocol. Then, DNA samples were stored at −20°C until single‐nucleotide polymorphism (SNP) analysis. Tetra‐primer amplification refractory mutation system–polymerase chain reaction (TP‐ARMS‐PCR) method was performed for genotyping of both *RORA* variants. The 25 μL PCR reaction contained 1 μL genomic DNA (100‐500 ng), 10 μL Taq DNA Polymerase 2X Master Mix Red (Amplicon, Denmark), 1 μL (10 pmol) of inner primers, 0.5 μL (5 pmol) of outer primers, and 11 μL PCR‐grade water. Amplifications were performed on a Biometra Thermal Cycler (Analytik Jena, Germany) according to the following cycling condition: initial denaturation at 94°C for 3 minutes, a subsequent series of 34 cycles of denaturation at 94°C for 30 seconds, annealing temperature as in Table 1 for 30 seconds, and extension at 72°C for 30 seconds. Final elongation was carried out at 72°C for 3 minutes. The primers were designed by using Primer1 online tool (Table 2).^28^ Amplified products were subjected to 2% agarose gel electrophoresis prepared in 0.5X TBE. The rs4774388 C and T alleles generated a 197 and 273 bp PCR products, respectively, and the outer primers amplified a common 412 bp amplicon. The rs11639084 T and C alleles produced 215 and 162 bp amplification products, respectively, and the outer primers amplified a common321 bp product. For further genotype confirmation, samples were randomly selected and sequenced by using an ABI 3730 × l DNA analyzer (Macrogen, Korea).

**Table 2:** Primer sequences and TP‐ARMS‐PCR conditions for rs4774388 and rs11639084

SNP	Primer	Primer Sequence	Amplicon Size (bp)	Ta (°C)
rs4774388	FO	CTGTGAGACCCTTTGACAACAGTACGTG	412	
	RO	TGAGGAAGAGTCCTTAGGAAGGGATGTC		65
	FI	CGGCATGATATTCATCGTCAAATCTGTTGC	197 (C allele)	
	RI	GCTGAGATGGAGACATCACAGAATGTCA	273 (T allele)	
rs11639084	FO	GTATTTGCATTTGTCATCCTTATCAACC	321	
	RO	AACTGTGGGCAAGTTATTTAACCTCTCT		57
	FI	GTTGCCAGCTAATGTTTATTGCATAATC	162 (C allele)	
	RI	TGGAGGCTTTAGTCTCTGGAACATATTA	215 (T allele)	

Note

The nucleotide specificity is indicated in parentheses.

Abbreviations: F, forward; I, inner; O, outer; R, reverse; SNP, Single‐nucleotide polymorphism; Ta, annealing temperature.

### 2.3 Statistical analysis

Genotype and allele frequencies and deviation from Hardy‐Weinberg equilibrium (HWE) were assessed by a chi‐squared test using SNPStats (available from http://bioinfo.iconcologia.net/SNPstats. The association between rs4774388 and rs11639084 and migraine was investigated in dominant and recessive inheritance modes. Odds ratios (ORs) with 95% confidence intervals (CI) were used to estimate the strength of association between the studied variants and migraine susceptibility. A *P‐*value of less than 0.05 was considered to be statistically significant.

## 3 RESULTS

**Figure 1:**
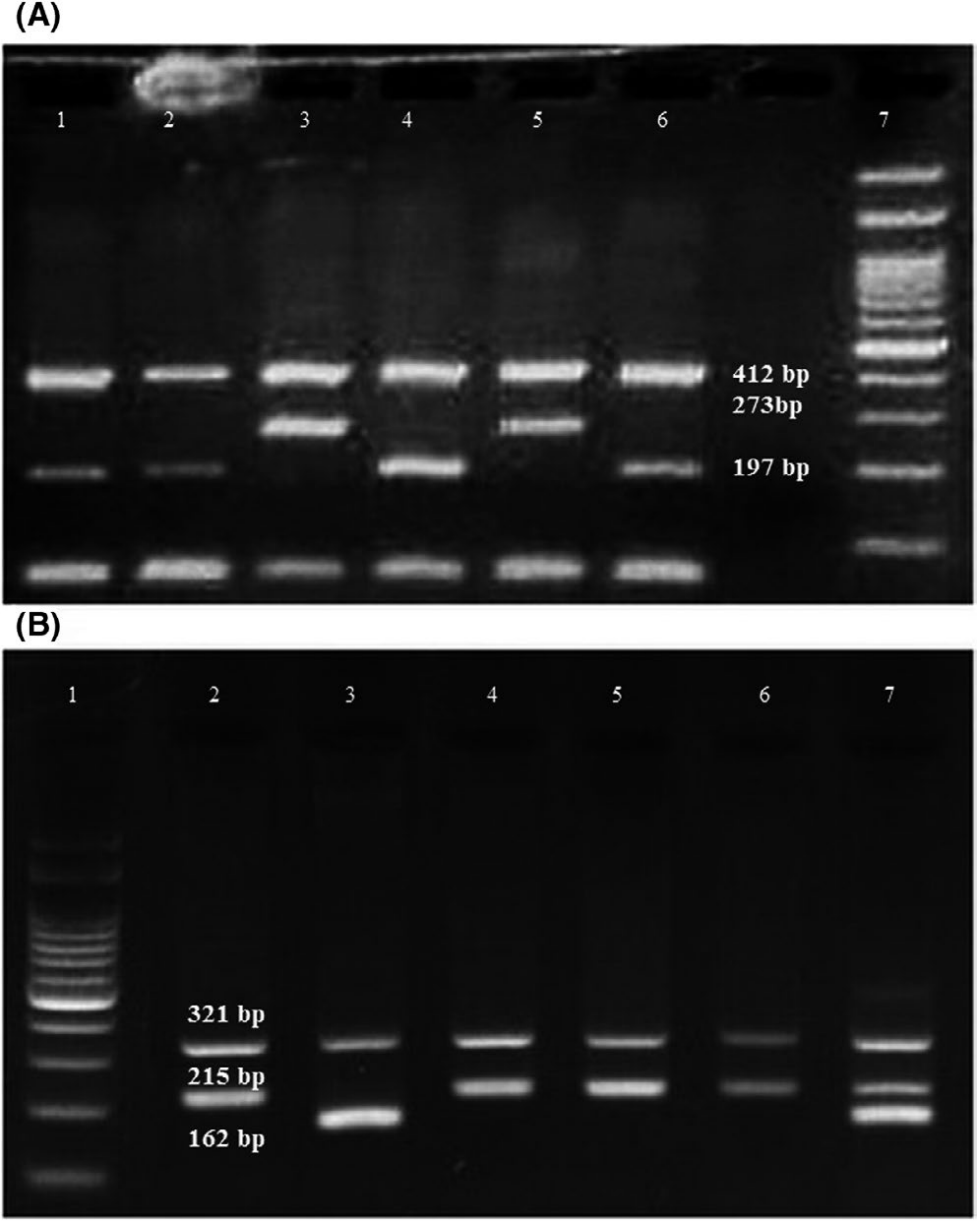
A, A representative 2% agarose gel electrophoresis for the identification of the RORA rs4774388 genotypes. Lanes 1, 2, 4, and 6: CC genotype; Lanes 3 and 5: TT genotype; Lane 7:100 bp DNA ladder. B, A representative 2% agarose gel electrophoresis for the identification of the RORA rs11639084 genotypes. Lane 1:100 bp DNA ladder; Lanes 2, 4, 5 and 6: TT genotype; Lane 3: CC genotype; lane 7: TC genotype

**Figure 2:**
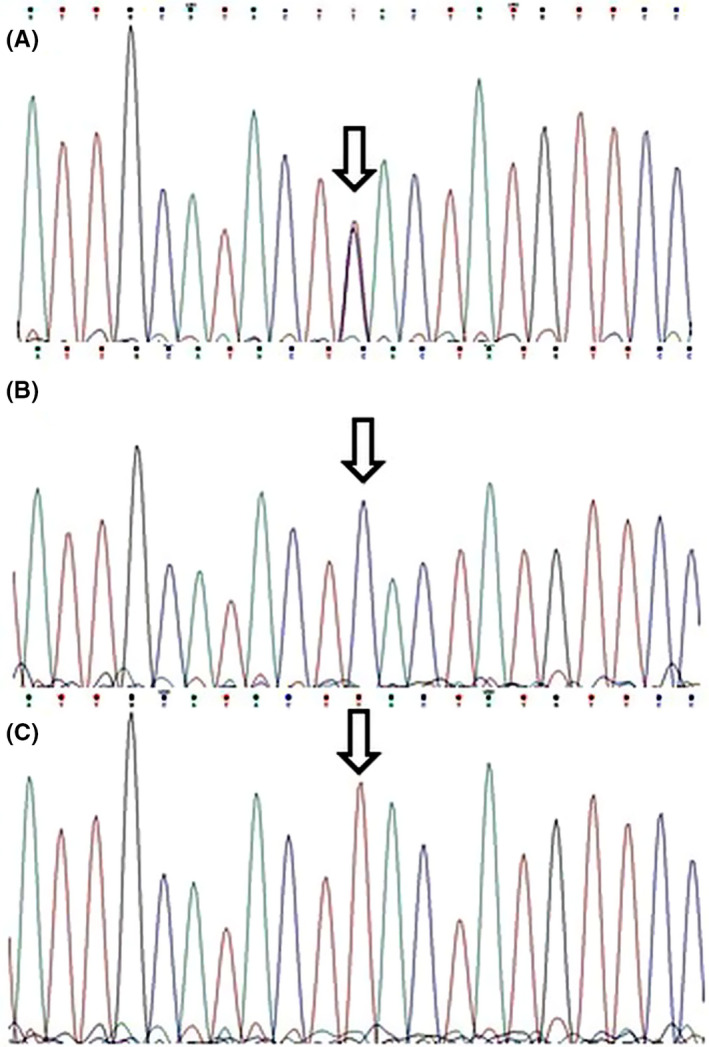
Electropherograms showing rs4774388 genotypes: A, CT; B, CC; C, TT. Arrows indicate the polymorphism locations

**Figure 3:**
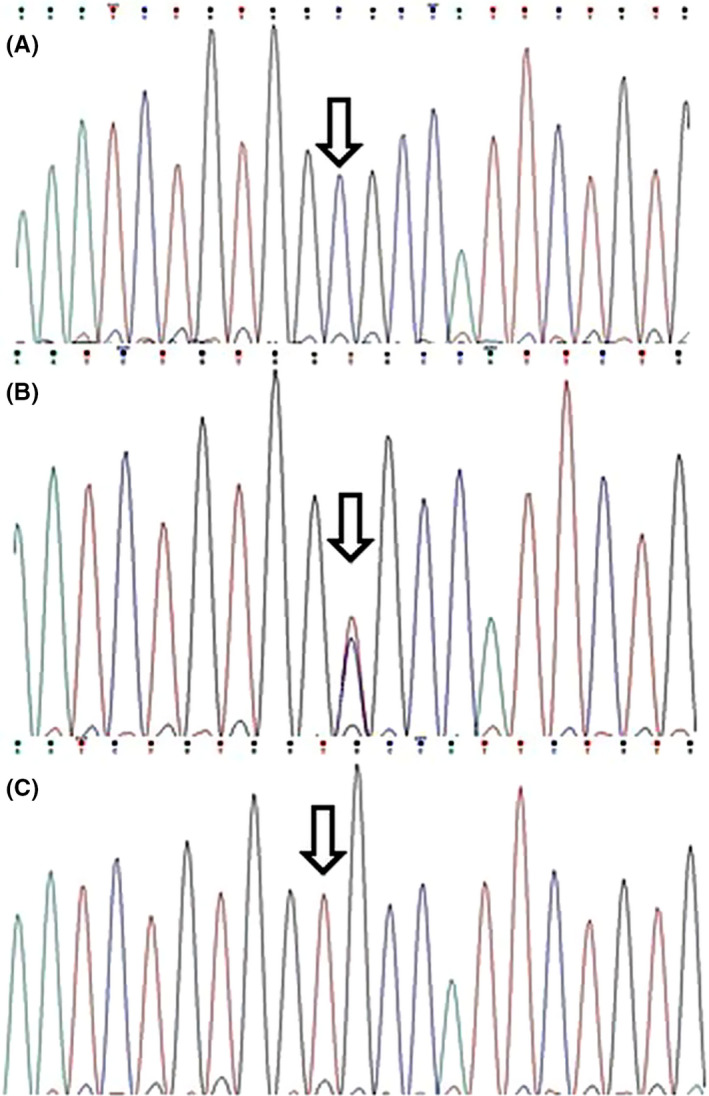
Electropherograms showing rs11639084 genotypes: A, CC; B, TC; C, TT. Arrows indicate the polymorphism locations

Figure 1 represents the agarose gels for genotyping the studied polymorphisms. *RORA* rs4774388 genotypic distribution was in complete HWE in the controls and patients; however, the control group deviated from HWE regarding rs11639084. Table 3 describes the genotype and allele frequencies for rs4774388 and rs11639084 polymorphisms in both migraineurs and healthy controls. The distribution of rs4774388 C/T and T/T genotypes differed significantly between the migraineurs and controls (*P* = 0.01 and *P* = 0.02, respectively). Moreover, rs4774388 was associated with migraine under a recessive model (*P* = 0.002; OR = 1.89.; CI = 1.25‐2.87). Genotype and allele frequencies for rs11639084 did not differ significantly between the studied groups. Figures 2 and 3 describe sequencing electropherograms for genotyping rs4774388 and rs11639084, respectively.

**Table 3:** Genotype and allele distribution for rs4774388 and rs11639084 in migraineurs and controls

Genotype	Migraineurs n (%)	Controls n (%)	OR (95% CI)	*P*‐value
rs4774388				
C/C	143 (71.5)	114 (57)	1.00 (reference)	—
C/T	48 (24)	67 (33.5)	0.57 (0.37‐0.89)	0.01
T/T	9 (4.5)	19 (9.5)	0.38 (0.16‐0.87)	0.02
C/C and C/T vs T/T			2.23 (0.98‐5.05)	0.05
C/C vs T/T and C/T			1.89 (1.25‐2.87)	0.002
Allele				
C	334 (83.5)	295 (73.75)	1.00 (reference)	—
T	66 (16.5)	105 (26.25)	0.56 (0.39‐0.78)	0.0008
rs11639084				
T/T	112 (56)	103 (51.5)	1.00(reference)	—
T/C	68 (34)	67 (33.5)	0.93 (0.61‐1.44)	0.75
C/C	20 (10)	30 (15)	0.61 (0.33‐1.15)	0.13
T/T and T/C vs C/C			1.59 (0.87‐2.90)	0.13
T/T vs T/C and C/C			1.20 (0.81‐1.78)	0.37
T	292 (73)	273 (68.25)	1.00(reference)	—
C	108 (27)	127 (31.75)	0.80 (0.59‐1.08)	0.14

Note

RAR‐related orphan receptor (*RORA*) involves in regulation of inflammation and circadian rhythm that probably are deregulated in migraine. This study, for the first time, confirmed the association between RORA and migraine.

Abbreviations: CI, confidence interval; OR, odds ratio.

## 4 DISCUSSION

Despite the remarkable role of genetic factors in migraine development, very few genes are documented as the intrinsic susceptible locus for this disease.^3^ The advancements in our ability to identify these variants as well as the involved signaling pathways have led to an improved understanding of genomic landscapes in migraine and the novel therapeutic approaches.

In the present study, for the first time, we described that *RORA* rs4774388 is significantly related to the migraine susceptibility.

Due to their locations, SNPs might affect gene splicing, expression, and mRNA stability. According to the HaploReg (version 4.1),^29^ rs4774388 is an intronic SNP, which serves as a binding motif for NKX3‐2, CEBPA, TCF11, and POU2F2 transcription factors involved in neuronal differentiation, inflammatory response, and blood cell differentiation.^30^, ^31^ Rs11639048 is located at the upstream of *RORA* gene and alters the binding affinity for BATF and NKX2 transcription factors. As suggested by Schraml et al, BATF plays an essential role in the differentiation of Th17 cells.^32^

*RORA* may contribute to migraine susceptibility and some symptoms through the regulation of Th17‐cell differentiation of and the circadian rhythm. In recent years, growing evidence has highlighted the role of inflammation in the pathophysiology of migraine. Previous studies revealed the alteration in the serum levels of anti‐ and pro‐inflammatory cytokines during migraine attacks. Accordingly, these are mostly including TNFα, IL‐6, and IL‐1β; however, contradictory observations also exist.^33^, ^34^, ^35^, ^36^

*RORA* plays a vital role in Th17‐cell differentiation and therefore regulates the secretion of IL‐17, suggesting a potential pro‐inflammatory role.^24^ Moreover, as a new subset of CD4^+^ T lymphocytes, Th17 cells secrete IL‐17 pro‐inflammatory cytokine family that promotes inflammation. IL‐17 is an important player in several autoimmune diseases, including rheumatoid arthritis and systemic lupus erythematosus. Recently, Hueber et al described that mast cells (MCs), but not Th17 cells, are the primary sources for the secretion of IL‐17A in rheumatoid arthritis synovium.^37^ This was an interesting finding since it is suggested that these cells may be involved in neuroinflammation in several neurodegenerative disorders, including cerebral ischemia, depression, neuropathic pain, autism, multiple sclerosis, and migraine.^38^, ^39^ Moreover, epidemiological studies have confirmed the association between MC‐related disorders such as asthma and migraine. Onur Turan et al suggested that migraine has a higher prevalence rate in asthma patients.^6^, ^7^ It may be more interesting when we know that high levels of IL‐17 are associated with severe asthma.^40^ Altogether, these observations further support the idea that migraine and MC‐related disorders have a common immune mechanism, which may be through the IL‐17 function.

Besides the regulation of cytokine‐mediated signaling pathway and inflammatory response, RORA seems to regulate several migraine‐related pathways, including circadian rhythm, cellular response to hypoxia, nitric oxide biosynthesis, and VEGF production.^8^, ^9^, ^10^, ^24^, ^41^

Several studies suggested that migraine attacks are under the control of the circadian rhythm and, as a result, occur more likely in the early morning.^42^, ^43^ Also, it is suggested that there is a bidirectional relationship between migraine and sleep disorders that more confirms the role of circadian rhythm impairment in this disease.^44^ RORA, as the nuclear receptor for melatonin, can explain deregulation of chronotype in migraineurs through the mediation of melatonin effect. Melatonin regulates the circadian rhythms in different manners. It is suggested that this circadian hormone can modulate the expressions of *BMAL1* and *CLOCK* as the main circadian clock genes. Furthermore, *RORA* and its repressor, *REV‐ERB*, are downstream targets for *BMAL1* and *CLOCK*.^45^ More importantly, melatonin contributes to the regulation of inflammation and neurodegeneration via RORA, and increases the expression of *RORA* itself. The interaction between clock genes and melatonin can also result in the deregulation of inflammatory cytokine levels, including TNFα, IL‐6, and IL‐1β.^45^ According to the He et al, RORA‐melatonin axis signaling pathway is important in myocardial ischemia, as one of the problems in migraine patients.^46^, ^47^

**Figure 4:**
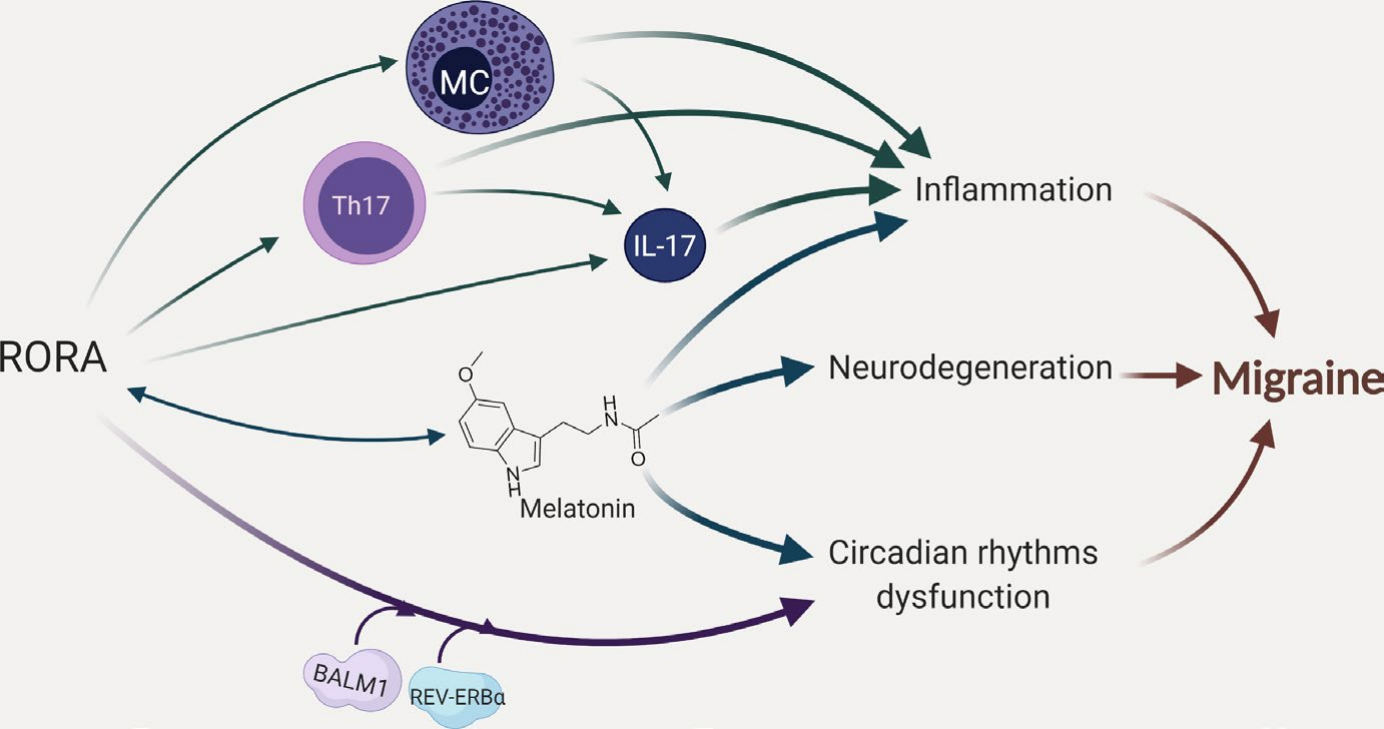
Schematic representation of the possible roles for *RORA* in migraine pathogenesis. *RORA* may contribute to migraine susceptibility as well as some symptoms with different mechanisms as follows: (i) affecting the expression of IL‐17 in Th17 and mast cells suggesting a pro‐inflammatory role; (ii) regulation of inflammation, neurodegeneration, and circadian rhythms via the mediation of melatonin effect; and (iii) affecting the circadian rhythms through the regulatory role of *BMAL1* and *REV‐ERB* on its expression

Several immune cells and immunological processes are under the control of the circadian‐related genes, and therefore, there is a link between inflammation and the biological clock.^48^, ^49^ In this regard, MCs are the most important and, as we mentioned earlier, play vital roles in migraine pathogenesis. Previous studies confirmed that MCs have a functional molecular clock and the expression of the related regulatory genes, including *CLOCK* and *BMAL1*obey from this circadian rhythm.^49^, ^50^ It may be interesting to know that RORA, as a transcription activator, can regulate the expressions of both *BMAL1* and its repressor *REV‐ERBα*.^22^ Figure 4 shows a schematic overview of the possible roles of RORA in migraine pathogenesis.

Considering the crucial role of *RORA* in the regulation of inflammation and circadian rhythm, it would be a good therapeutic target in migraine treatment. Up to now, several natural and synthetic ligands are detected and also designed for RORA that harbor agonist or antagonist effects. T0901317 and SR3335 serve as *RORA* modulators, which reduce the expressions of its target genes by suppressing the gene transcriptional function.^18^ SR1001, as the derivative of T0901317, suppresses the differentiation of Th17 cells by selective targeting RORA, and hence, it may be used in the treatment of the Th17‐mediated diseases.^51^

## 5 CONCLUSION

Current results suggest *RORA* may serve as a molecular link between inflammation and circadian rhythm dysfunction in migraine. Therefore, we recommend RORA as a new susceptible locus in migraine, which may offer new therapeutic intervention. Further research considering the role of *RORA* in migraine may enhance our understanding of the mechanisms underlying this gene.

## CONFLICT OF INTEREST

The authors declare no conflicts of interest in this work.

## Data Availability

The data that support the findings of this study are available from the corresponding author upon reasonable request.
